# Neurolinguistic measures of typological effects in multilingual transfer: introducing an ERP methodology

**DOI:** 10.3389/fpsyg.2015.01087

**Published:** 2015-08-07

**Authors:** Jason Rothman, José Alemán Bañón, Jorge González Alonso

**Affiliations:** ^1^School of Psychology and Clinical Language Sciences, University of ReadingReading, UK; ^2^Department of Language and Linguistics, UiT The Arctic University of NorwayTromso, Norway; ^3^Basque Center on Cognition, Brain and LanguageDonostia-San Sebastian, Spain; ^4^Department of English and German Philology, University of the Basque CountryVitoria, Spain

**Keywords:** third language (L3) acquisition, transfer, event-related potentials (ERPs), agreement, artificial language

## Abstract

This article has two main objectives. First, we offer an introduction to the subfield of generative third language (L3) acquisition. Concerned primarily with modeling initial stages transfer of morphosyntax, one goal of this program is to show how initial stages L3 data make significant contributions toward a better understanding of how the mind represents language and how (cognitive) economy constrains acquisition processes more generally. Our second objective is to argue for and demonstrate how this subfield will benefit from a neuro/psycholinguistic methodological approach, such as event-related potential experiments, to complement the claims currently made on the basis of exclusively behavioral experiments.

## Introduction

Empirical investigations into adult multilingual acquisition have been done for decades and from a multitude of paradigms (see De Angelis, 2007; Edwards and Dewaele, 2007; Rothman et al., 2013 for review). Prior to the last decade or so, it was not obvious that the study of a third or more languages in adulthood should constitute its own subfield of acquisition research, that is, distinct from the study of a non-native second language (L2). As Edwards and Dewaele (2007, p. 221) state, there is a “growing awareness that trilingualism is not just an extension of bilingualism,” meaning that the idea that studying multilingualism simply presents more of the same as bilingualism no longer prevails. It is now definitively clear that there are methodological, cognitive, linguistic, and epistemological reasons why L3 acquisition must be considered independently (see e.g., De Angelis, 2007; cf. de Bot and Jaensch, 2015).

With few exceptions, for example Klein (1995), studies on L3 acquisition of morphosyntax from a formal linguistic perspective did not emerge until the early 2000s. Since then there has been a sharp increase of interest and output of research in adult multilingual acquisition within the generative tradition (see Leung, 2007; Rothman et al., 2011). As pointed out by García Mayo and Rothman (2012), to date much of this work has focused on investigating previous language transfer source(s)^1^ under the mindset that doing so is relevant to and provides unique evidence for litigious questions that concern all acquisition research. For example, investigating how transfer—influence from previously acquired mental linguistic representations—is constrained in adult multilingualism, where several potential options/sources are available, ultimately contributes to a more fine-grained understanding of underlying linguistic representations and the role of cognitive economy in acquisition processes more generally (see Flynn et al., 2004; Rothman, 2013, 2015 for details).

At present three formal models of L3/Ln morphosyntactic transfer have proved influential in spawning what can now be considered an emerging subfield of generative L3 transfer studies. Not surprisingly given the paradigm in which they are conceived, each of these models is predicated on the notion that multilingual acquisition in adulthood is subject to universal constraints and that transfer in multilingualism is not at all random, but rather is delimited by linguistic and/or cognitive factors. These three models, to be reviewed in greater detail in Section “L3 Models of Morphosyntactic Transfer,” are: (i) the *L2 Status Factor* (Bardel and Falk, 2007, 2012; Falk and Bardel, 2011), (ii) the *Cumulative Enhancement Model* (CEM, Flynn et al., 2004; Berkes and Flynn, 2012) and (iii) the *Typological Primacy Model* (TPM, Rothman, 2010, 2011, 2013, 2015). A commonality between them is the shared belief that adult learners are able to acquire new morphosyntactic representations^2^ past puberty and that more than strictly speaking linguistic variables (i.e., cognitive considerations) contribute to what ultimately determines selection of transfer and even its timing. Yet, differences in their proposals result in mutually exclusive predictions that render them empirically falsifiable against one another.

Some experimental studies have offered data that are compatible with more than one of these models. This is not surprising since these models do not always offer incompatible predictions depending on the language triad and order of acquisition of the languages under investigation. In the body of this paper, we will introduce and discuss much of the existing empirical data, offering some insights into what we believe they tell us when coupled together. In doing so, we will address the first of two goals of this paper, which is to introduce the reader to this emerging field and the empirical evidence it provides. Since the existing data come exclusively from behavioral methodologies, the second goal of this paper is to show how the methodological remit of generative L3 studies can be expanded to include neurolinguistic methodologies such as event-related potentials (ERPs), as has been done in recent generative L2 work (e.g., Gabriele et al., 2013a; Alemán Bañón et al., 2014). To this end, we will detail how these models make clear predictions that can be tested with an ERP methodology, and articulate a sample methodology we contend is suitable to test these predictions.

## L3 Models of Morphosyntactic Transfer

In the past decade, three generative L3/Ln models of morphosyntactic transfer have been proposed. This section introduces these models, which we propose are testable against one another via processing methodologies, such as ERP.

### The L2 Status Factor

As the name suggests, the L2 Status Factor is a model of multilingual transfer which assigns a privileged role to the L2 at the initial stages of L3 acquisition (e.g., Bardel and Falk, 2007; Falk and Bardel, 2011). It is argued that the L1 is not as accessible as the L2 for transfer, presumably because the L2 is represented and stored in a different memory system (declarative memory), relative to the L1 (procedural memory). Falk and Bardel (2011) and Bardel and Falk (2012) adopt a synthesis of Ullman’s (2001, 2005) and Paradis’ (2004, 2009) Declarative/Procedural (DP) models of bilingualism to offer what they claim to be a neurolinguistic basis for the L2 Status Factor.

The question of why L3 learners would default to suppressing the L1 and rely more heavily on the L2 is of great epistemological importance for the L2 Status Factor. Bardel and Falk (2012) argue that doing so is essentially a byproduct of assumed cognitive similarity between the L2 and the L3. They claim that both the L2 and L3 differ from L1 grammars in terms of the developmental path, the degree of ultimate attainment, and the memory systems they draw from (declarative vs. procedural). In DP models, the grammar of the L1 is sustained by procedural memory (implicit), while declarative or lexical memory (explicit) supports both the L1 lexicon and, at least at the initial stages, the grammar of all late-acquired languages (i.e., L2, L3, Ln). Bardel and Falk (2012) adopt the DP divide of L1 vs. L2 representation and argue that it results in bypassing the L1 as a primary or even possible source of transfer in L3 acquisition.

The data that best support the L2 Status Factor come from Bardel and Falk (2007) and Falk and Bardel (2011). Bardel and Falk (2007) examined placement of negation in two different groups: L1 V2^3^/L2 non-V2 and L1 non-V2/L2 V2, learning either Swedish or Dutch as an L3, both of which are V2 languages. Their data showed that the L1 non-V2/L2 Dutch/German group outperformed the L1 V2/L2 English group in producing post-verbal negation. They maintained that only a privileged role for the L2 is corroborated by the data. Despite compelling evidence that typology was not necessarily a deterministic factor, one must keep in mind that these learners are not beginners and that what we observe could actually be a byproduct of L3 interlanguage development itself. That is, it is possible that the pattern would have been distinct if the learners had been tested at an earlier, more appropriate stage in L3 development for the question of transfer source.

Despite plenty of data that clearly show that the L2 is a potential source of L3 transfer, there are less data that unambiguously support the L2 Status Factor’s principled claim that it should be the privileged or only source. That is, much of the data showing that the L2 is transferred is not in a position to preclude other variables, such as typological similarity or maximal facilitation, as being the actual deterministic factors for the selection of the L2. The L2 Status factor is clear: despite other variables that might favor the L1 from a typological or facilitative point of view, the L2 should be chosen, precisely due to the neurocognitive reasons detailed above, as cited by Bardel and Falk (2012). Just like showing L1 transfer would only be consistent with absolute transfer under certain methodologies and language pairings, demonstrating L2 transfer might only be consistent with the possibility of L2 transfer as opposed to falsifying alternative explanations. Rothman and Cabrelli Amaro (2010) mention this in their study, which examined properties related to the Null Subject Parameter in L3 French and L3 Italian. Their study could be cited as strong support for the L2 Status Factor insofar as their data show L2 transfer and are thus entirely consistent with the L2 Status Factor’s predictions. However, Rothman and Cabrelli Amaro (2010) ultimately concluded that they were unable to differentiate between an L2 Status Factor effect and possible (psycho)typological influences, since the choice of L2 and L3 in their methodology conflated both variables (i.e., English was always the L1, Spanish was always the L2, and the L3 was either French or Italian). This same confound is not true of Bardel and Falk (2007) and Falk and Bardel (2011), so it is interesting that they show a very strong L2 effect despite apparent structural proximities between the L3 and the L1. Nevertheless, a number of studies call into question the absolute position of L2 transfer, thus rendering the steadfast line of the L2 Status Factor problematic (e.g., Na Ranong and Leung, 2009; Hermas, 2010; Iverson, 2010; Rothman, 2010, 2011; Montrul et al., 2011; Giancaspro et al., 2015; Slabakova and García Mayo, 2015).

It might be suggested that L2 transfer even under this approach can be circumvented by structural or other factors, which Bardel and Falk do not deny in their published work (see for example Falk et al., 2015^4^). However, it seems unclear how this would be possible under the current explanation based on a DP difference between the L1 and other grammars and the hypothesized suppression of the L1 that this creates. In other words, it is not clear how or why factors such as relative structural similarity could bypass the filter imposed by purported cognitive differences (reliance on declarative vs. procedural memory) related to the L1 and L2.

### The Cumulative Enhancement Model

The CEM proposed by Flynn et al. (2004) posits that both the L1 and the L2 are possible sources of morphosyntactic transfer at the initial stages of L3 acquisition. The CEM maintains that language acquisition is a collective process throughout the lifespan whereby experience with the acquisition of any prior language can facilitate subsequent language acquisition. Differently from the L2 Status Factor, the CEM claims that previous linguistic knowledge transfers in multilingual development from any language available to the learner, irrespective of order of acquisition. However, transfer crucially only obtains when such knowledge has a facilitative effect, since language acquisition is assumed to be a non-redundant process. Alternatively, when transfer from either language would not be facilitative it is effectively blocked.

Flynn et al. (2004) base their claims on data from the production of restrictive relative clauses in L1 Kazakh/L2 Russian/L3 English speakers. Their data demonstrate that experience in any previously acquired language can be taken advantage of, providing support for the CEM. Still, there has not been much published work that supports the CEM unambiguously (but see Jaensch, 2011; Berkes and Flynn, 2012, for claims of support for a ‘weak’ version of the CEM; see also Slabakova and García Mayo, 2015, for a discussion of the roles of cumulative enhancement and its interaction with cumulative inhibition).

Supported by a growing literature, as we will see in greater detail below, is the CEM’s claim that transfer is not restricted to a default L1 or default L2. Amassing evidence in the generative L3 transfer literature supports the CEM’s claim that acquisition is inherently non-redundant by cognitive design. Conversely, the strong claim that non-facilitative transfer cannot obtain is simply not supported by much of the available evidence. The evidence reviewed above related to the L2 Status Factor already demonstrates counter evidence to such a claim. Clear motivations for why the CEM rejects non-facilitative transfer as a possibility remain elusive. From our perspective, having to avoid non-facilitative transfer *a priori* places an unrealistic burden on limited cognitive resources during the formation of the L3/Ln system. At a minimum, it implies that the learner would have to have enough experience with the L3/Ln on a property-by-property basis to determine what could be facilitative, and also to suppress what would be non-facilitative even when strong evidence of overall structural similarity between two of the grammars is overwhelming. It also seems to suggest that transfer is incremental throughout L3 development. As such, both the L1 and the L2 would need to remain equally activated throughout the L3 process, which entails a cognitive cost that creates a burden on finite resources.

### The Typological Primacy Model

The TPM (Rothman, 2010, 2011, 2013, 2015) is a model of L3/Ln transfer that, similar to the CEM, envisions access to both the L1 and L2 mental grammars at the initial stages. Differently from the CEM, however, the TPM acknowledges the possibility of non-facilitative transfer, which derives from the same general spirit underlying the original CEM: for reasons of general cognitive economy, language acquisition is forced to be a non-redundant process. Both the CEM and the TPM agree that multilingualism is conditioned by a cumulative effect of previous linguistic acquisition; however, the TPM views selection of a language for transfer as being conditioned by factors related to underlying structural similarity between the languages at play, as opposed to mere facilitation.

Recall that for the CEM, transfer at the initial stages and beyond is predicted to be maximally facilitative or otherwise neutralized. Unlike the CEM, the TPM hypothesizes that transfer is complete (the entire L1 or L2) and early in L3 interlanguage development, and is determined by the structural similarity between the target L3 and the L1 or L2, as assessed by the internal (linguistic) parser. More precisely, it makes reference to structural similarities at an underlying level of linguistic competence across the three languages. Therefore, the possibility of non-facilitative transfer is taken not only to be possible, like the L2 Status Factor (albeit for different reasons), but rather predictable.

**FIGURE 1:**
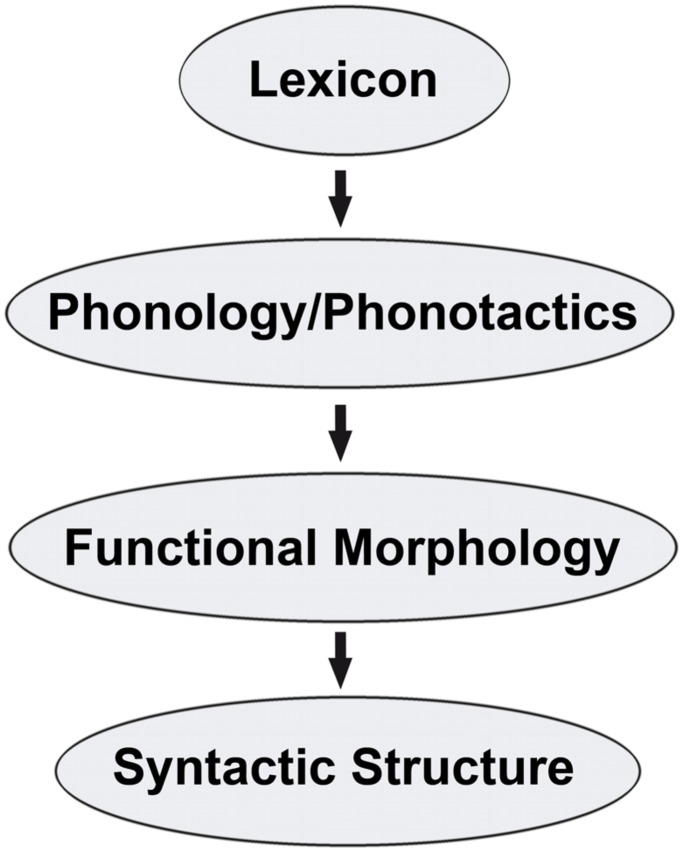
**Implicational hierarchy of input cues.** Adapted from Rothman (2013, 2015).

Proposals for how the linguistic parser determines at an early stage whether the L1 or L2 should transfer have been the topic of recent work (Rothman, 2013, 2015). Following the logic advocated in Schwartz and Sprouse’s (1996) Full Transfer/Full Access Hypothesis for L2 acquisition, the TPM advances the idea that one of the two systems must be transferred completely in the initial stages. A continuum of cues related to four factors is hypothesized to lead the parser to select between the two available grammars, represented in **Figure 1**.

Not all of these factors are as easily usable by or equally accessible to the parser at the same time, partially depending on the specific language pairings. For this reason, the above list is intended to be implicationally hierarchical. The TPM does not idealize an unrealistic situation in which these four factors are mutually exclusive to one another. Rather, there is clear mutual dependency of the levels in the hierarchy. For example, syntactic structure clearly depends on functional morphology, which in turn is determined in the lexicon and interfaces with phonology. Rothman (2013) makes it clear that, of the four possible types of cues, it is ultimately the language combinations themselves that determine how many and which, if any, of the four factors are usable. Ultimately the TPM predicts that the previously acquired linguistic system with the most detectable/usable structural crossover, at the highest levels of the cue hierarchy, at the earliest of timing at the very initial stages of L3 will be selected for complete transfer.

Now let us turn our attention to the empirical evidence in support of the TPM. Rothman (2010) examined the L3 acquisition of Brazilian Portuguese, contrasting two sets of L3 learners: (a) L1 speakers of English who were highly proficient learners of L2 Spanish and (b) L1 speakers of Spanish who were highly proficient learners of L2 English. The study examined word order restrictions relating to transitive verbs and two types of intransitive verbs (unergatives and unaccusatives) in declaratives and interrogatives, as well as relative clause attachment preference. Despite the fact that Spanish and Brazilian Portuguese are typologically similar, Brazilian Portuguese patterns much more like English than Spanish in these related domains. The data unambiguously show Spanish transfer irrespective of whether it was an L1 or L2, supporting the TPM and providing evidence against the predictions of the L2 Status Factor and the CEM.

In recent years, several studies have shown that relative structural similarity between the L3 and one of the previously acquired systems is the most deterministic factor for multilingual transfer. Much of the additional work supporting the typological factor in adult multilingualism comes from language triads where two Romance languages and English are involved (e.g., Foote, 2009; Iverson, 2009, 2010; Ionin et al., 2011; Montrul et al., 2011; Borg, 2013; Giancaspro et al., 2015). This fact might leave one questioning whether the TPM makes predictions beyond such obvious language pairings in the Romance family (see Rothman, 2015). If the TPM is on the right track, predictions should be derivable irrespective of the languages implicated in any triad. Rothman’s (2013, 2015) articulation of the TPM claims that it makes universal predictions. Promisingly, recent research with more varied L3 language pairings has shown similar support for the TPM (e.g., L1 Tuvan/L2 Russian/L3 English, Kulundary and Gabriele, 2012; L1 Uzbek/L2 Russian/L3 Turkish, Özçelik, 2013; L1 Polish/L2 French/L3 English, Wrembel, 2012; L1 English/L2 Spanish/L3 Arabic, Goodenkauf and Herschensohn, 2014).

For example, Özçelik (2013) examined the L3 acquisition of Turkish by Uzbek-Russian bilinguals with respect to quantificational scope. For ease of exposition, we will use English to explain the linguistic facts. Whereas Uzbek (similar to English) has both surface and inverse scope interpretations of sentences like (1), Turkish only has the surface scope interpretation (2).

(1) Jack didn’t find two guys.
✓ (a) Surface: It is not the case that Jack found two guys. (Jack found one guy, three guys, no guys, etc.)
✓ (b) Inverse: There are two guys that Jack didn’t find.
(2) Jack iki kişi bul-ma-dı.
  Jack two person find-NEG-PAST
  “Jack didn’t find two guys.”
✓ (a) Surface: It is not the case that Jack found two guys. (Jack found one guy, three guys, no guys, etc.)
✕ (b) Inverse: There are two guys that Jack didn’t find.

The L3 acquisition of Turkish by Uzbek–Russian bilinguals in this regard is interesting in that, although Turkish and Uzbek are both Turkic languages and are typologically related, Turkish behaves like Russian with respect to this structure, and differently from Uzbek, which allows both scope interpretations. The results show that the learners treat Turkish like Uzbek, as they allow both surface and inverse scope interpretations of sentences like (2), i.e., they transfer from the holistically TYPOLOGICALLY similar language (Uzbek), rather than from Russian, the language that is STRUCTURALLY similar to Turkish for this particular property. Results support the TPM, as transfer is activated on the basis of overall typological similarity, even though this leads to a less optimal grammar since the source language for transfer (Uzbek) and the target language (Turkish) behave differently with respect to the construction tested here and despite the fact that Russian, the L2, would have been more facilitative for this property.

## EEG and the ERP Methodology: Use and Application to L3

To date, all of the experimentation done under the current models of L3/Ln transfer has been methodologically behavioral. Although illuminating, we will argue that these models also make predictions that can be tested with online methodologies, such as ERP. We argue that testing these predictions can add new insights to and strengthen the descriptive and explanatory power of these models.

### EEG and ERPs

EEG is an electrophysiological method that records at the scalp the electrical activity generated by large populations of neurons firing in synchrony. It provides high temporal resolution, with millisecond precision, and therefore it is an excellent tool to examine the dynamics of language processing as it unfolds over time. However, unlike methods such as functional magnetic resonance imaging (fMRI) or positron emission tomography (PET), EEG provides limited spatial resolution, due to the fact that the signal recorded at the scalp cannot be unambiguously traced back to its source (Friederici, 2004). Event-related potentials (ERPs) are small voltage changes that are time-locked to a specific event of interest. For example, if the event of interest is agreement resolution, we can time-lock the EEG signal to the element in the sentence where the parser can determine whether or not agreement was successful (e.g., *Harold saw this house/^∗^houses yesterday*). If a comparison across conditions (e.g., grammatical vs. ungrammatical) reveals differences in the morphology of the waveforms, we can assume that the brain was sensitive to the property under investigation. One clear advantage of ERPs is their multidimensional nature. ERPs can be examined in terms of their latency (the time window when the effect emerges), amplitude (the strength of the effect), and polarity (whether the voltage change is negative or positive). They can also be examined in terms of their scalp topography (the electrode region or regions where the effect is captured). Importantly, this allows for a very in-depth characterization of the mechanisms underlying language processing and for a very fine-grained comparison between different populations (e.g., native speakers vs. adult language learners). One of the most unique advantages of the ERP methodology is the fact that different ERP components, such as the N400 and the P600, are modulated by different aspects of language processing. The P600 (e.g., Osterhout and Holcomb, 1992; Hagoort et al., 1993) is a positive deflection between 500 and 900 ms whose elicitation is attributed to processes of syntactic reanalysis (e.g., Osterhout and Holcomb, 1992; Gouvea et al., 2010), syntactic integration (e.g., Kaan et al., 2000), and syntactic repair (Hagoort et al., 1993; Osterhout and Mobley, 1995). While not all processes which affect the P600 are syntactic (or even linguistic) in nature, it is noteworthy that this is the only component that is consistently found for syntactic agreement violations in native speakers (e.g., Coulson et al., 1998; Gunter et al., 2000; Hagoort, 2003; Wicha et al., 2004; Barber and Carreiras, 2005; Martín-Loeches et al., 2006; Nevins et al., 2007; Frenck-Mestre et al., 2008; O’Rourke and Van Petten, 2011), making it the most reliable ERP signature associated with the native processing of syntactic agreement.

In contrast, the N400 is a negative-going wave between 200 and 600 ms which typically emerges in central posterior electrodes of the EEG cap and which has been found to be sensitive to the strength of lexical associations (see Kutas and Federmeier, 2011 for a review). For example, words that are semantically associated with a previously presented prime (e.g., *dog-cat*) show reduced N400 amplitudes relative to words unrelated to the prime (e.g., *car-pen*) (Holcomb and Neville, 1990). Studies on native processing where the only ERP signature associated with syntactic agreement violations is the N400 are rare. One exception is Barber and Carreiras (2005), who examined number and gender violations in Spanish word pairs, and found a larger N400 for both violation types relative to grammatical strings. Since isolated word pairs do not require syntactic structure building, Barber and Carreiras (2005) interpret these findings as evidence that the Spanish native speakers processed the agreement violations at the lexical level, by comparing the lexical features of the agreeing words. Interestingly, when the exact same violations were examined in sentences, they yielded a P600.

In a subset of studies, the P600 is preceded by a negative-going wave in the N400 time window, sometimes with a left anterior distribution. The qualitative nature of this negativity is very much a matter of debate. Some authors have identified it as the Left Anterior Negativity (LAN), a component argued to index automatic morphosyntactic processing (e.g., Friederici et al., 1996). A problem with this interpretation, however, is that a number of studies examining morphosyntactic processing in native speakers do not find the LAN for agreement errors (e.g., Wicha et al., 2004; Frenck-Mestre et al., 2008; Alemán Bañón et al., 2012). Alternatively, this negativity has been identified as an N400. Under this interpretation, the left anterior distribution of the N400 results from its topographical overlap with a central-posterior P600, which cancels out the negativity in central-posterior regions of the scalp (e.g., Guajardo and Wicha, 2014; Tanner and Van Hell, 2014). Under this view, the N400 is argued to reflect either the semantic integration difficulty caused by the presence of the agreement error (e.g., Guajardo and Wicha, 2014), or individual differences with respect to processing strategies, with some individuals relying on lexical information (N400) and others on combinatorial information (P600) (Tanner, 2013, 2015; Tanner and Van Hell, 2014). Importantly for the purposes of the present study, it is the P600 that consistently emerges for morphosyntactic errors in native speakers, even if sometimes it is preceded by a negativity. The reverse, however, is not true. As stated in Tanner (2015), agreement errors in native speakers are unlikely to yield an N400 not followed by the P600:

“(…) given the dominance of P600 effects in response to morphosyntactic violations across individuals, it is highly unlikely to randomly draw a sample of individuals where only a reliable N400 would be found, with no following P600 — even though some individuals show negativity-dominant brain responses to morphosyntactic violations.”

(Tanner, 2015, p.154).

### ERP and Formal Linguistic Approaches to SLA

How can we use the ERP methodology to test formal linguistic theoretical models of adult language acquisition? To give one example, Alemán Bañón et al. (2014) relied on the difference between the N400 and the P600 to adjudicate between the Full Transfer/Full Access Hypothesis (Schwartz and Sprouse, 1996) and the Interpretability Hypothesis (Tsimpli and Dimitrakopoulou, 2007; see also Gabriele et al., 2013a). The study examined the processing of number and gender agreement in L2 Spanish by advanced English-speaking learners. Critically, these two hypotheses differ with respect to whether or not adult L2 learners are predicted to be able to show native-like processing for novel uninterpretable features (in this case, Spanish gender agreement). Only the Full Transfer/Full Access Hypothesis predicts so, since L2 acquisition is hypothesized to be influenced but not constrained by the properties of the L1 (e.g., White et al., 2004).

Under the Interpretability Hypothesis, in contrast, English-speaking learners of Spanish are not predicted to show native-like processing for gender agreement, regardless of proficiency. Learners might exhibit behavior that appears native-like (e.g., high accuracy rates in behavioral tasks; see Franceschina, 2005 for an example), but the supporters of the Interpretability Hypothesis argue that such behavior is achieved through compensatory strategies (e.g., Hawkins, 2001). For example, learners might establish associations between morphemes that tend to co-occur, in which case gender violations might yield a larger N400 than grammatical sentences (similar to what Barber and Carreiras, 2005, found for word pairs in Spanish native speakers). Alternatively, learners might rely on the phonological similarity between the agreeing words (in Spanish, most masculine nouns end in –*o* and most feminine nouns end in –*a*), in which case gender violations should only modulate the N400 component, consistent with a number of studies which have examined the effects of phonological similarity on word processing^5^.

Alemán Bañón et al.’s (2014) proposal is that if English-speaking learners of Spanish can process novel features in a native-like manner, they should show a P600 for gender violations, consistent with a large body of literature which reports P600 effects for agreement violations in native speakers (including the Spanish-speaking controls reported in Alemán Bañón et al., 2012, 2014, for whom this was the only component found for number and gender violations across the different syntactic contexts tested). However, if learners rely on other mechanisms, such as comparing the lexical features of the agreeing words or relying on their phonological similarity (as would be predicted by the Interpretability Hypothesis), gender violations should yield a larger N400 than grammatical sentences (e.g., Barber and Carreiras, 2005; Coch et al., 2008). The advanced L1 English L2 Spanish learners in Alemán Bañón et al. (2014) showed robust P600 effects (and no N400) for both number and gender violations overall. This evidence was used to argue that native-like processing for features that are unique to the L2 is possible in adult L2 acquisition, consistent with full UG accessibility in adulthood. These results are also consistent with previous ERP studies providing evidence that, at an advanced level of proficiency, adult learners can exhibit native-like processing for L2 morphosyntactic properties (e.g., Rossi et al., 2006), including those that are not instantiated in the L1 (e.g., Dowens et al., 2010, 2011; Foucart and Frenck-Mestre, 2012). What is most relevant about the approach by Alemán Bañón et al. (2014) is that it shows how the ERP methodology can be used to shed light on the qualitative nature of L2 processing and, more importantly for the present discussion, to test current theoretical models of adult language acquisition.

In another relevant study, Bond et al. (2011) found a P600 for both number and gender violations in adult English-speaking learners of Spanish at a lower level of proficiency. Interestingly, the L2 learners also showed a larger P600 for number (present in the L1) than gender (unique to the L2) violations, which is consistent with the possibility that, at lower levels of proficiency, processing is more heavily impacted by L1 transfer (e.g., Tokowicz and MacWhinney, 2005; see Dowens et al., 2010, and Foucart and Frenck-Mestre, 2011, for further evidence for transfer effects in advanced learners).

Importantly for the present discussion, ERP has also been used to examine the initial stages of L2 processing. For example, McLaughlin et al. (2010) tracked L1 English learners throughout their first year of university L2 French. The linguistic focus of the study was subject-verb agreement, which is instantiated in both English and French, and article-noun number agreement, which is only instantiated in French. For subject-verb agreement violations, a subset of “fast” learners (*n* = 7) showed an N400 effect (violations being more negative than grammatical sentences) after only 1 month of instruction, which the authors interpret as evidence that learners were sensitive to the violations but did not process them grammatically from the start. After 4 and 6 months of instruction, however, the same violations yielded a P600 (similar to the native controls). Article-noun number violations, in contrast, did not yield any effects at any point. In light of these results, McLaughlin et al. (2010) argue against full transfer in the initial stages, since learners did not show evidence of grammatical processing for the property that was available through the L1 (subject-verb number). Instead, the authors propose that learners initially treat all grammatical violations at the lexical level by relying on co-occurrence frequencies between morphemes (e.g., pronouns and verbal inflection; see also Ullman, 2001, 2005).

The results by McLaughlin et al. (2010) are not supported by another longitudinal study by Gabriele et al. (2013b). The authors examined morphosyntactic development in novice English-speaking learners of Spanish. The study focused on three types of agreement: (1) subject-verb number, which is realized in both English and Spanish, (2) noun-adjective number, which is only morphologically realized in Spanish, and (3) noun-adjective gender, which is unique to Spanish. In native speakers, all violation types yielded robust P600 effects (Bond et al., 2011). Interestingly, the learners (*n* = 23) showed a small positivity in the P600 time window for both types of number violations (feature that is present in the L1) after only 2 months of instruction. Crucially, after 6 months of instruction, this positivity became more robust and showed a broader scalp distribution, more in line with the canonical P600 elicited by the Spanish controls. Gender violations, in contrast, yielded neither N400 nor P600 effects at any point. Since the learners showed sensitivity (a positivity) to the feature that is shared by the L1 and L2 (number) after only 2 months of instruction, Gabriele et al. (2013b) argue in support of theories that assign a privileged role to the properties of the L1 at the initial stages.

The above studies provide very relevant findings for our goal of using ERP to examine the initial stages of L3/Ln acquisition. The logic is as follows: if L2ers show ERP signatures akin to native speakers for a given grammatical property, then we can assume that, in principle, the property at stake is available as a source of transfer. If so, we might expect that advanced L1 English L2 Spanish bilinguals learning Portuguese as an L3 might show a positivity in the P600 time window for both number and gender violations in Portuguese. Showing this for gender would make them different from the English-speaking learners of Spanish reported in Gabriele et al. (2013b), who only showed this positivity for number. Such findings would be consistent with the TPM and the CEM (for different reasons), but crucially not with the L2 Status Factor. Recall that, under the current formulation of the L2 Status Factor, the L2 and L3 are hypothesized to be stored in declarative memory. As stated in Ullman (2001, 2005), learners’ greater reliance on declarative memory is predicted to yield N400 effects for grammatical violations where native speakers show qualitatively different components (e.g., a biphasic LAN-P600 pattern according to Ullman, 2001). Therefore, if the L2 Status Factor is on the right track, novice learners of L3 Portuguese whose L1 and L2 are English and Spanish, respectively, should show, at most, N400 effects for gender agreement violations in L3 Portuguese. This is one example of how the ERP methodology (i.e., the fact that the N400 and the P600 have been argued to be associated with different aspects of processing and different memory systems) can be used to adjudicate between the above models in a way that behavioral methodologies cannot. With respect to the CEM and the TPM, since transfer by either facilitation (CEM) or by typological proximity (TPM) would always favor Spanish transfer, there is no way to tease apart these models with the present domain of grammar. In Section “Sample ERP Methodology,” we will provide a sample methodology that is able to tease apart all three initial stages models.

## Sample ERP Methodology

In order to test the above models of L3 acquisition, we detail a novel methodology that is part of our in progress work, which relies on the use of artificial languages (AL) as L3s and which combines behavioral and processing measures (i.e., grammaticality judgment task and ERP data). The use of ALs offers two crucial advantages. First, we can test truly *ab initio* learners, allowing us to better contrast the predictions of the above models, all of which are initial stages models. Second, by using ALs we can systematically manipulate the similarity between the L3 and the L1/L2 in terms of (1) the presence/absence of a given feature and (2) the levels of the cue hierarchy which, according to Rothman (2013, 2015), will determine the parser’s selection of a transfer source. In addition, the use of ERP will shed light on the qualitative nature of processing at L3 initial stages. This is especially relevant, given the current articulations of the L3 models under review. For example, the L2 Status Factor (Bardel and Falk, 2012) argues that L3 acquisition relies mainly on declarative memory and, therefore, L3 beginners are predicted to show N400 responses for morphosyntactic properties associated with qualitatively different components in native speakers (e.g., P600 or a biphasic LAN-P600; e.g., Ullman, 2001; Morgan-Short et al., 2012). In contrast, the TPM assumes that the initial state of L3 acquisition is the entire L1 or L2 and, therefore, this model predicts that “transferable” morphosyntactic properties should be associated with ERP signatures that are qualitatively native-like from the start (e.g., P600; Rothman, 2015).

The linguistic focus of the proposed study is number and gender agreement. This choice is motivated on the basis that most previous ERP studies looking at the initial stages of L2 processing have focused on this domain (e.g., Osterhout et al., 2006; Morgan-Short et al., 2010; Gabriele et al., 2013b). Therefore, we can make predictions regarding the initial stages of L3 processing based on our knowledge of how agreement in processed at the initial stages of L2 acquisition. In addition, our study could provide insight into the differences and similarities between the L2 and L3 acquisition of these grammatical properties. Our rationale is based on two core findings: (1) The longitudinal study by Gabriele et al. (2013b) looking at L1 English beginners of L2 Spanish shows ERP signatures consistent with transfer of grammatical number (present in the learners’ L1) from the earliest of stages tested; (2) A number of studies have shown native-like ERP signatures for grammatical gender in advanced L1 English learners (e.g., Dowens et al., 2010, 2011; Foucart and Frenck-Mestre, 2012; Gabriele et al., 2013a; Alemán Bañón et al., 2014). From (1) we believe it reasonable to use ERP to examine transfer at the initial stages of L3 acquisition. Furthermore, (2) suggests that, for the acquisition of an L3 that realizes gender agreement, we can predict sensitivity to gender not only in L3ers who are L1 Spanish-L2 English, but also in L3ers who are L1 English-L2 Spanish (provided they have reached a high level of proficiency in L2 Spanish). If both groups show sensitivity to grammatical gender in the L3, this would immediately call into question the L2 Status Factor (especially if brain responses are not in the form of N400 effects, which is the component argued to be associated with declarative memory).

Recall, however, that—for the above learning scenario—both the CEM and the TPM predict the transfer of gender irrespective of L1/L2 sequencing. The two models differ in the conditions under which this transfer should happen. Under the TPM, the learner’s perceived similarity between the L3 and the L1/L2 will determine the source of transfer. Under the CEM, gender will be transferred when appropriate, based on the fact that it has already been acquired in a previous language (Spanish). Our design contrasts the predictions of these two models by using two ALs as L3s. One of the ALs is lexically similar to English (“Mini-English”) and the other one, to Spanish (“Mini-Spanish”), but they both instantiate number and gender agreement. This lexical similarity between English and Mini-English should have a non-facilitative effect under the TPM (i.e., the parser should assume that Mini-English does not instantiate gender based on the fact that English does not realize this property). Under the CEM, this negative transfer should be blocked, and the parser will transfer gender from the facilitative language, Spanish.

### Artificial Languages

Following work by Williams and colleagues (e.g., Williams, 2004; Williams and Kuribara, 2008; Marsden et al., 2013), Mini-English is built on the English lexicon and novel morphemes for number and gender have been added to articles and adjectives. The second AL, Mini-Spanish, is based on the Spanish lexicon where also completely novel morphemes for number and gender have been added to articles and adjectives. Each AL includes 12 inanimate nouns (six masculine, six feminine) and 12 adjectives, in order to facilitate the learning of its lexicon. Each AL also includes one article that inflects for number and gender (four variants: masculine-singular, feminine-singular, masculine-plural, feminine-plural), one copulative verb that inflects for number (singular, plural), one conjunction, one adverb, and two locatives. Since one of our research questions concerns the role of lexical similarity on the selection of a transfer source, all other potential cues are neutralized in the ALs. For example, training in the AL will take place in the visual modality (as opposed to aural), to avoid providing phonological information. Likewise, learners will only be exposed to meaningful examples of the AL where word order is similar in English and Spanish, in order to neutralize word order as a cue. Examples of short sentences in Mini-Spanish are provided in (3) and (4) below:

(3)
  (a) Ne **camion** es car-enu.
    the_-MASC-SG_ truck is expensive_-MASC-SG_
  (b) Ner **camion** son car-enur.
    the_-MASC-PL_ truck are expensive_-MASC-PL_
  (c) Ge **llave** es car-egu.
    the_-FEM-SG_ key is expensive_-FEM-SG_
  (d) Ger **llave** son car-egur.
    the_-FEM-PL_ key are expensive_-FEM-PL_

(4)
  (a) Ge **llave** es sobre ne reloj.
    the key is above the watch.
  (b) Ge **llave** es bajo ne reloj.
    the key is below the watch

As can be seen in (3a-b), the masculine noun *camion* “truck,” which has been selected from the Spanish lexicon, must agree in number and gender with the preceding article (masculine-singular: *ne*; masculine-plural: *ner*) and the predicative adjective (masculine-singular: *carenu*; masculine-plural: *carenur*). A similar example is provided in (3c-d), where the feminine noun *llave* “key,” also from the Spanish lexicon, agrees in number and gender with the preceding article (feminine-singular: *ge*; feminine-plural: *ger*) and the predicative adjective (feminine-singular: *caregu*; feminine-plural: *caregur*). All of the nouns in Mini-Spanish have the same lexical gender as their Spanish counterparts. Importantly, all nouns have been selected such that, despite their lexical similarity with their equivalent in Spanish, they do not exhibit the markers typically associated with the masculine/feminine distinction in Spanish (e.g., masculine –*o*, feminine –*a*), to avoid providing learners with additional morphological cues. Notice also that, similar to Morgan-Short et al.’s (2010) study, the nouns *camion* and *llave* provide no phonological cues regarding the gender of the noun. This was done in an attempt to prevent learners from relying on a purely phonological strategy when computing gender agreement. In order for the comparison between number and gender to be more ecologically valid, nouns in the ALs are also opaque for number, as shown in (3a-b) and (3c-d). The sentences in (4) show the distribution of the locatives “above” and “below” in Mini-Spanish. With respect to the design of Mini-English, semantically equivalent nouns and adjectives were used (e.g., *truck, key*). With respect to lexical gender, since English lacks this property altogether, we decided to assign Mini-English nouns the same lexical gender as the nouns in Mini-Spanish (i.e., *truck* and *key* are masculine and feminine, respectively, similar to *camion* and *llave*). Examples of mini-English are provided in (5) and (6) below:

(5)
  (a) Ne **truck** is expens-enu.
    the_-MASC-SG_ truck is expensive_-MASC-SG_
  (b) Ner **truck** are expens-enur.
    the_-MASC-PL_ truck are expensive_-MASC-PL_
  (c) Ge **key** is expens-egu.
    the_-FEM-SG_ key is expensive_-FEM-SG_
  (d) Ger **key** are expens-egur.
    the_-FEM-PL_ key are expensive_-FEM-PL_

(6)
  (a) Ge **key** is above ne watch.
    the key is above the watch.
  (b) Ge **key** is below ne watch.
    the key is below the watch

The structure of interest will be the agreement relation between the noun and the predicative adjective, which will be located across a verb phrase (VP; e.g., *the truck_V P_*[*is expensive*]). Although it has been argued that agreement relations are more taxing when they are non-local (i.e., across a verb phrase) for both native speakers (e.g., Alemán Bañón et al., 2012) and L2 learners at an advanced level of proficiency (Foucart and Frenck-Mestre, 2012; Alemán Bañón et al., 2014), our choice is motivated upon the grounds that this is a syntactic context where English and Spanish exhibit similar word order (e.g., *el camión es caro* “the truck is expensive”). In contrast, when agreement is local, the position of the adjective with respect to the noun differs in English and Spanish (e.g., *camión caro* “truck expensive”). We are justified in restricting the design of the study to lexical similarity given Rothman’s (2013, 2015) claims regarding the primacy of the lexicon for determining transfer [see The Typological Primacy Model (1) above]. Indeed, this is sufficient to test between the three models, which is the primary goal of our study. To further test the very claim of primacy of the lexicon over actual syntactic cues made by the TPM, the next methodological step would be to offer additional competing cues in the ALs. For example, adding to Mini-English a syntactic property that conflicts with the English grammar but is grammatical in Spanish would allow us to test the TPM cue hierarchy independently, since we would have a case where the lexical level is similar to English, but the morphological and syntactic levels are similar to Spanish. The TPM is clear: the lexical level, which is argued to be the most detectable one and, therefore, the top level of the hierarchy, should neutralize the use of the other cues.

### Participants

With respect to the participants, our study includes four groups of English-Spanish bilinguals who differ along two criteria: (1) the order of acquisition of English vs. Spanish, and (2) the AL they will be trained on. All L3 learners will have acquired their L2 after ∼11 years of age and will have high-proficiency in the L2. After the completion of the L3 study, all learners will be tested in their L2 for knowledge of the relevant properties (i.e., agreement). This is to ensure that the relevant properties are in place in the L2 and can, therefore, transfer to the L3. **Table 1** below offers a schematic of the learner groups in our design.

**Table 1:** Breakdown of groups based on L1-L2-AL combination.

Group	L1	L2	Languages of training
Group 1 (*N* = 24)	English	Spanish	Mini-Spanish
Group 2 (*N* = 24)	English	Spanish	Mini-English
Group 3 (*N* = 24)	Spanish	English	Mini-Spanish
Group 4 (*N* = 24)	Spanish	English	Mini-English

### Artificial Language Training

The study involves a training session in the AL and a judgment task with an EEG recording. During the training, learners will be exposed to meaningful examples of the AL. No metalinguistic explanations are provided, to ensure training is implicit (e.g., Morgan-Short et al., 2010). The training simulates a picture-sentence matching task (e.g., Mueller et al., 2005). Learners see two pictures showing a contrast (e.g., 3 expensive trucks vs. 3 cheap trucks) and their written description in the AL (e.g., “The trucks are expensive” vs. “The trucks are cheap”). By using both masculine and feminine nouns, both in the singular and in the plural, L3 learners receive implicit input on number and gender agreement between articles, nouns, and adjectives. The training will start with simple article-noun phrases and then move to full sentences like the ones in (3) and (4) above. Filler items will be included which manipulate the location of a noun with respect to another noun, via the locatives “above” and “below.” Each noun and adjective is presented an equal number of times throughout the training. The same amount of meaningful examples is provided for number and gender. Learners are exposed to 272 meaningful examples (68 per number/gender combination).

To ensure that learners attend to the training, they will complete a comprehension quiz at the end. Learners see a picture (e.g., 3 cheap trucks) and must select the sentence in the AL that best describes it from among five options. Alongside the correct description of the picture (“The trucks are cheap”), the options include a sentence with a violation of gender agreement, a sentence with a number violation, and a sentence with a double violation (number and gender). In half of the items the violation is realized between the article and the noun and, in the other half, between the noun and the adjective. As a control, the fifth option involves a semantic violation (e.g., “The trucks are expensive”), to ensure that learners are able to extract meaning from the pictures used in the AL training. Filler items involve pictures which manipulate the location of two nouns (e.g., a key above a watch). Here, the possible responses include a sentence that correctly describes the picture (“The key is above the watch”), and four incongruent sentences. Two of the incongruent sentences involve the use of the wrong locative (e.g., “The key is below the watch,” “The watch is above the key”) and the other two involve the use of incorrect nouns. Upon providing their response, learners receive a “correct” or “incorrect” message, which is visually displayed on the computer screen. No other feedback is provided, to ensure that training in the AL remains as implicit as possible. The quiz includes an equal number of sentences with masculine and feminine nouns, and an equal number of sentences with singular and plural nouns. Each noun and adjective is tested an equal number of times throughout the quiz.

Learners are graduated from the training once they reach above chance accuracy in the quiz, which is defined as the ratio of correct responses to the total number of responses (i.e., 20% accuracy). Learners who score below this threshold must take the training again. This necessarily means that different learners will receive different amounts of training, but it ensures that learners have achieved approximately the same level of proficiency at the time of the EEG recording.

### Grammaticality Judgment Task

For the purposes of this task, the 12 nouns in each AL have been crossed with the 12 adjectives, yielding a total of 144 noun-adjective combinations. Those agreement dependencies have been embedded in sentences like the one in (7) below, which has six different versions. The sentence structure where we manipulate agreement is based on a previous study on number and gender agreement in Spanish by Alemán Bañón et al. (2012, 2014). Examples are provided for a sentence with a masculine noun in Mini-Spanish.

(7)
  (a) Ne **camion** es car-enu y ne reloj tambien.
    the_-MASC-SG_ truck_-MASC-SG_ is expensive_-MASC-SG_ and the watch too
  (b) Ne **camion** es ^∗^car-enur y ne reloj tambien.
    the_-MASC-SG_ truck_-MASC-SG_ is expensive_-MASC-PL_ and the watch too
  (c) Ne **camion** es ^∗^car-egu y ne reloj tambien.
    the_-MASC-SG_ truck_-MASC-SG_ is expensive_-FEM-SG_ and the watch too
  (d) Ner **camion** son car-enur y ner reloj tambien
    the_-MASC-PL_ truck_-MASC-PL_ are expensive_-MASC-PL_ and the watches too
  (e) Ner **camion** son ^∗^car-enu y ner reloj tambien
    the_-MASC-PL_ truck_-MASC-PL_ are expensive_-MASC-SG_ and the watches too
  (f) Ner **camion** son ^∗^car-egur y ner reloj tambien
    the_-MASC-PL_ truck_-MASC-PL_ are expensive_-FEM-PL_ and the watches too

Each one of the 144 sentences will be assigned to one of three conditions: grammatical (7a,d), number violation (7b,e), or gender violation (7c,f). An equal number of masculine and feminine nouns will be used. Likewise, the study involves an equal number of singular and plural nouns. Learners will read the 144 sentences presented one word at a time using the Rapid Serial Visual Presentation Method (RSVP; SOA: 450/300 ms; Alemán Bañón et al., 2012, 2014) while their brain activity is recorded with EEG. There will be 48 items per condition, which corresponds to the mean number of trials per condition reported in Molinaro et al.’s (2011) review of ERP studies on agreement. As can be seen in (7), the adjective is never sentence-final, to avoid semantic wrap-up effects that have been observed in final position (e.g., Hagoort, 2003). At the end of each trial, learners will perform a grammaticality judgment task (e.g., Mueller et al., 2005; Morgan-Short et al., 2010). The motivation for using a grammaticality judgment is twofold. First, having information regarding the learners’ accuracy will allow us to determine the extent to which learners detected the agreement violations at the behavioral level. Second, it has been argued that the amplitude of the P600 is sensitive to the explicitness of the task. As discussed in Molinaro et al. (2011), the amplitude of the P600 tends to decrease when native speakers are asked to read for meaning, as opposed to focus on grammatical correctness (although it should be noted that the P600 emerges even in the absence of a judgment task; see for example Hagoort et al., 1993). Therefore, since the population of interest involves novice L3 learners, where effects are not predicted to be quantitatively native-like or even robust, we believe it is more appropriate to use a grammaticality judgment task, similar to previous ERP L2 studies using the artificial language paradigm (e.g., Mueller et al., 2005; Morgan-Short et al., 2010).

An additional 96 grammatical fillers will be added to the experimental materials (a total of 240), in order to balance the number of grammatical and ungrammatical sentences in the design. Fillers manipulate the position of a given noun with respect to another noun (see the sentences in 4 and 6 above). Importantly, they do not include adjectives and, therefore, shift the attention away from noun-adjective agreement.

### Predictions

All three models predict that all learner groups should show sensitivity to number agreement, since both English and Spanish realize this property. It is for gender agreement that the three models make competing predictions. The L2 Status Factor makes two clear predictions: (1) since only the L2 should transfer, only the learner groups who have Spanish as the L2 (Groups 1 and 2) should show sensitivity to gender violations, even if the L3 being acquired is typologically different from L2 Spanish, as is the case for L1 English-L2 Spanish bilinguals trained in Mini-English; (2) brain responses should index reliance on the declarative memory system across the board, that is, number violations should yield N400 effects (with no evidence of a P600 at this stage) in all groups, and so should gender violations in Groups 1 and 2.

For the CEM, all groups should show qualitatively native-like responses to both number and gender (e.g., P600-like component, similar to the L1 English novice learners of Spanish in Gabriele et al., 2013b, which might be preceded by a negativity) since order of acquisition of Spanish should be inconsequential and such transfer would be facilitative^6^. For the TPM, only the groups who are trained in Mini-Spanish (Groups 1 and 3) should show sensitivity to gender violations, given considerations of the typological proximity of the languages. For Groups 2 and 4, the lexical similarity between Mini-English and English should mislead the parser into assuming Mini-English does not realize gender agreement. **Table 2** summarizes the predictions in terms of ERP signatures for number and gender agreement violations for all three models.

**Table 2:** Predicted ERP responses for number and gender agreement.

L1-L2-AL Combination	L2 Status Factor		CEM		TPM	
	Number	Gender	Number	Gender	Number	Gender
L1 English-L2 Spanish, L3: Mini-Spanish	N400	N400	(N400)-P600	(N400)-P600	(N400)-P600	(N400)-P600
L1 English-L2 Spanish, L3: Mini-English	N400	N400	(N400)-P600	(N400)-P600	(N400)-P600	
L1 Spanish-L2 English, L3: Mini-Spanish	N400		(N400)-P600	(N400)-P600	(N400)-P600	(N400)-P600
L1 Spanish-L2 English, L3: Mini-English	N400		(N400)-P600	(N400)-P600	(N400)-P600	

*We do not predict quantitatively native-like ERP components for any of the properties under examination in any of the groups (e.g., Gabriele et al., 2013b). We use the terms N400 and P600 to highlight the qualitative differences between the predicted effects. We use parentheses to indicate the possibility that the N400 preceding the P600 for agreement violations under the CEM and the TPM might not emerge.*

Behaviorally, the three models predict that all learner groups should perform above chance levels (i.e., above 50% accuracy) with number agreement, since both English and Spanish realize this property. With respect to gender agreement, the L2 Status Factor predicts that only Groups 1 and 2 (i.e., those with Spanish as the L2) should show above chance accuracy with the detection of gender violations. In contrast, the TPM predicts that only Groups 1 and 3 (i.e., those trained in Mini-Spanish) should show above chance performance with gender violations. Finally, the CEM predicts similar performance for number and gender across all groups.

This example methodology shows how obtaining ERP evidence for the multilingual transfer debate is possible and how its application to the literature dominated by behavioral methodology could add new insights.

## Conclusion

In this article, we hope to have shown how the ERP methodology can be used to further our understanding of the factors which impact multilingual transfer. After introducing the main theoretical models of L3 acquisition, we provided relevant evidence from existing ERP studies on the native and non-native processing of agreement which strongly motivates the use of ERP to examine transfer at the initial stages of L3 acquisition (i.e., the central question in all three models discussed). Most importantly, we articulated a methodology from our in progress work which combines the ERP methodology and the artificial language paradigm to examine L3 initial stages transfer and whose novelty resides in the fact that it can adjudicate between current articulations of the L2 Status Factor, the CEM, and the TPM in a way that behavioral methodologies cannot. Here, we focused on the domain of grammatical agreement, but it should be noted that the methodology can also be used to examine other domains of grammar, including those which have been investigated in previous L3 behavioral studies (e.g., word order). Enlightening as it is, evidence for and against the L2 Status Factor, the CEM and the TPM consists exclusively of oﬄine, behavioral data. Ideally, data from online methodologies, such as ERP, will complement what has been shown behaviorally and add new insights to these models. Corroborative or contradicting evidence from processing can strengthen the descriptive and explanatory power of these models or present novel data requiring refinements to them.

## Author Contributions

JR: The first author conceived the project, was involved in all aspects of the design of the proposed methodology, and contributed to the drafting of Sections “Introduction,” “L3 Models of Morphosyntactic Transfer,” and “Conclusion.” JAB: The second author conceived the project, was involved in all aspects of the design of the proposed methodology, and contributed to the drafting of Sections “EEG and the ERP Methodology: Use and Application to L3” and “Sample ERP Methodology,” and “Conclusion.” JGA: The third author was also substantially involved in all aspects of the design of the proposed methodology and critically revised the manuscript. All authors are responsible for final approval of the version to be published and agree to be accountable for all the aspects of the work in ensuring that questions related to the accuracy or integrity of any part of the work are appropriately investigated and resolved.

## Conflict of Interest Statement

The authors declare that the research was conducted in the absence of any commercial or financial relationships that could be construed as a potential conflict of interest.

## Footnotes

1 There is, of course, notable work in generative L3 studies that investigate interlanguage development, regressive transfer effects in development and competence at later stages of acquisition such as, for example, García Mayo et al. (2005), Cabrelli Amaro and Rothman (2010), García Mayo and Villarreal Olaizola (2011), Cabrelli Amaro (2013), Slabakova and García Mayo (2015).

2 Based on the most recent papers by Bardel and Falk (2012), in which they appeal to the so-called declarative/procedural distinction following Paradis (2004), it is no longer completely clear to us that what the L2 Status Factor takes as L2 mental linguistic representation is the same as the CEM and the TPM, the latter of which maintain a clear distinction between learned and acquired knowledge and exclusively focus on the latter type of L2 knowledge. See “L3 Models of Morphosyntactic Transfer” below for further discussion.

3 V2 refers to verb-second, a distinctive property of Germanic languages (except for English). In V2 languages, the finite verb appears in second position of a declarative main clause, whereby the first position is occupied by a single major constituent that functions as the clause topic. V2 languages do differ with respect to the distribution of the V2 rule, often referred to as micro-parametric variation: while some V2 languages restrict the V2 rule to matrix clauses (e.g., German, Dutch), others have V2 in matrix and subordinate clauses alike (e.g., Swedish, Norwegian).

4 In a recent paper, Falk et al. (2015) acknowledge that with certain populations typological relatedness might trump the L2 privilege. However, the authors are very clear that such a possibility only obtains in learners that are metalinguistically aware, even trained, in their L1 and L2, for example individuals who are trained teachers of their L1 as well as successful learners of an L2.

5 For example, words which are phonologically similar to their prime (e.g., *lake-break*) show a reduction in N400 amplitude compared to words that are phonologically unrelated to the prime (e.g., *lake-line*) (e.g., Coch et al., 2008).

6 As mentioned in Section “EEG and ERPs,”, some studies have reported a biphasic N400-P600 pattern for syntactic agreement errors in native speakers, and argued for individual differences in the processing of agreement, with most individuals showing a P600 and a subset of them showing an N400. We have, thus, incorporated in our predictions the possibility that the P600 might be preceded by an N400, but we note that most of the available evidence for individual differences in agreement processing comes from studies which have examined subject-verb agreement with English auxiliary verbs in designs which include lexical semantic violations, which are known to modulate the N400 (e.g., Tanner and Van Hell, 2014; Tanner et al., 2014). It remains an open question whether the same variability might emerge in designs that examine other features (i.e., gender) and other syntactic contexts (noun-adjective agreement), and which do not manipulate semantic congruency.
